# Insights into IL‐29: Emerging role in inflammatory autoimmune diseases

**DOI:** 10.1111/jcmm.14697

**Published:** 2019-10-02

**Authors:** Jia‐Min Wang, An‐Fang Huang, Wang‐Dong Xu, Lin‐Chong Su

**Affiliations:** ^1^ Department of Evidence‐Based Medicine School of Public Health Southwest Medical University Sichuan China; ^2^ Department of Rheumatology and Immunology Affiliated Hospital of Southwest Medical University Sichuan China; ^3^ Department of Rheumatology and Immunology Minda Hospital of Hubei Minzu University Enshi China

**Keywords:** autoimmunity, inflammation, interleukin‐29, signalling

## Abstract

Interleukin‐29 (IL‐29) is a newly discovered member of type III interferon. It mediates signal transduction via binding to its receptor complex and activates downstream signalling pathways, and therefore induces the generation of inflammatory components. Recent studies reported that expression of IL‐29 is dysregulated in inflammatory autoimmune diseases, such as rheumatoid arthritis, systemic lupus erythematosus, osteoarthritis, Sjögren's syndrome, psoriasis and systemic sclerosis. Furthermore, functional analysis revealed that IL‐29 may involve in the pathogenesis of the inflammatory autoimmune disorders. In this review, we will systematically review the current knowledge about IL‐29. The information collected revealed the regulatory role of IL‐29 and may give important implications for its potential in clinical treatment.

## 1 INTRODUCTION

The newly discovered cytokine interleukin‐29 (IL‐29) belongs to type III interferon (IFN), which has another two subfamilies IL‐28A and IL‐28B. They are also known as IFN‐λ1, IFN‐λ2 and IFN‐λ3, respectively.^1^ The gene encoding IL‐29 owns 5 exons and locates on the long arm of human chromosome 19.^2^ These mediators are members of a large family called IL‐10 family, composed of nine cytokines: IL‐10, IL‐19, IL‐20, IL‐22, IL‐24, IL‐26, IL‐28A, IL‐28B and IL‐29.^3^, ^4^ All type III IFNs bind to the same receptor complex consisting of IFN‐LR1 (also named IL‐28R1, CRF2‐12 or LICR) and IL‐10R2 (IL‐10R2 and CRF2‐4).^5^, ^6^ Recently, IL‐29 has been focused much attention. Production of IL‐29 was found specific to some tissues, and there was a tissue specificity responding to IL‐29.^7^ IL‐29 receptor is expressed in dendritic cells, T cells, intestinal epithelial cells and leukaemia cells. The important role of IL‐29 in tumours and its potential use for clinical therapy has been widely discussed.^8^ Due to antiviral and immunoregulatory characteristics of IL‐29, studies also showed that IL‐29 performed significantly in the pathogenesis of inflammatory autoimmune diseases,^9^ for instance, systemic lupus erythematosus (SLE),^10^ rheumatoid arthritis (RA),^11^ psoriasis^12^ and Sjögren's syndrome (SS).^13^ The discovery of type III IFNs opens up a new field of IFN research in which IL‐29 is considered to be a core member. On account of the strong association between this molecule and inflammatory autoimmune diseases, we systematically review recently published articles on this significant relationship. The regulatory capacity of IL‐29 in inflammatory autoimmune diseases has drawn increased attention to these studies. It is hoped that the information collected will contribute to future research on IL‐29, and may provide some clues for its role in inflammatory autoimmune diseases. Furthermore, our review may give important implications for its potential in clinical treatment.

## 2 IL‐29 SIGNALLING

**Figure 1:**
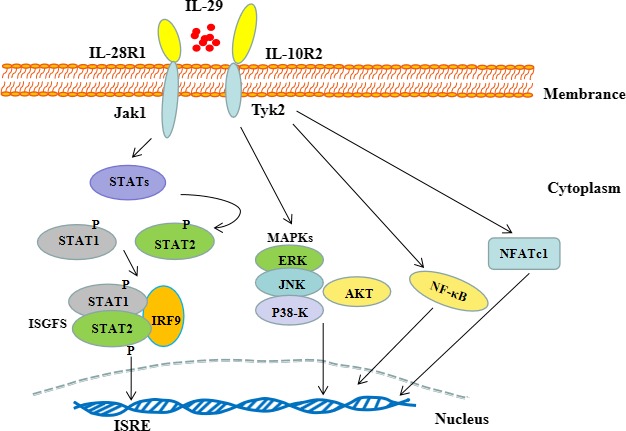
Signal transduction pathways initiated by IL‐29. IL‐29, a heterodimeric cytokine, binds to receptor complex composed of IL‐28R1 and IL‐10R2. The induced Janus kinase/signal transduction and activator of transcription (JAK‐STAT) pathway leads to the activation of STAT1 and STAT2, and subsequently combines with IRF9 to form ISGF3 transcription factor complex. The formed complex transmits signalling to the nucleus. Similarly, the activating signalling was transmitted to the nucleus via mitogen‐activated protein kinase (MAPK), protein kinase B (Akt), nuclear factor‐kappa B (NF‐κB) and nuclear factor of activated T cell 1 (NFATc1) pathways, presenting the ability of antiviral protection, anti‐proliferative response and immune regulation

Studies suggested that IL‐29 is able to activate downstream signalling pathways, and therefore induces the generation of inflammatory components. Activation of Janus kinase/signal transduction and activator of transcription (JAK‐STAT) signalling pathway might be induced by IL‐29 through STAT1 and STAT2. Similarly, activation of STAT3 and STAT5 by IL‐29 also proved to affect the JAK‐STAT signalling.^7^, ^14^ Consistently, IL‐29 induced signal transduction through activation of protein kinase B (Akt) and mitogen‐activated protein kinase (MAPK). It is notable that the ability of IL‐29 to affect signalling pathways may depend on specific cells. Osteoarthritis (OA) fibroblast‐like synovial cells (FLS) stimulated with IL‐29 activated JAK‐STAT, MAPK and Akt signalling pathways, resulted in phosphorylation of the proteins.^15^ Mast cell line P815 treated with IL‐29 promoted the production of IL‐4 and IL‐13 through phosphatidylinositol 3‐kinase (PI3K)/Akt and JAK‐STAT3 signalling pathways.^16^ Monocyte‐derived macrophage responded to IL‐29 treatment via STAT1 phosphorylation.^17^ In RAW264.7 cells, an elevation of lipopolysaccharide (LPS)‐induced nuclear factor‐kappa B (NF‐κB) signalling activation was observed following IL‐29 stimulation.^11^ In addition, IL‐29 down‐regulated expression of nuclear factor of activated T cell 1 (NFATC1)–mediated osteoclastogenic genes such as tartrate‐resistant acid phosphatase (TRAP), cathepsin K (CTSK) and matrix metalloprotein 9 (MMP‐9) through activation of c‐Jun N‐terminal kinase (JNK), and inhibition of c‐Fos, and NFATc1 in receptor activator of nuclear factor‐κ B ligand (RANKL)‐stimulated RAW264.7 cells.^9^ Moreover, IL‐29 regulated toll‐like receptor 3 (TLR3) expression in keratinocytes, but it was hindered following adding JAK inhibitor 1, suggesting that IL‐29–induced TLR3 generation may depend on the activation of JAK‐STAT pathway.^18^ It is recognized that bone erosion in RA correlated with increased production of pro‐inflammatory cytokines and accelerated osteoclastogenesis in affected joints. IL‐29 suppressed osteoclastogenesis by activation of STAT signalling pathway and inhibition of NF‐κB activation, and NFATc1 translocation.^9^ When tyrosine residues on STATs were phosphorylated, some homodimers and heterodimers were formed and translocated into nucleus, and then combined with IFN‐stimulated response elements (ISREs) in regulatory regions of the IFN‐stimulated genes (ISGs). For example, ISG factor 3 (ISGF3), a transcription complex, consisted of phosphorylated STAT1, STAT2 and IFN regulatory factor (IRF) 9, initiated the transcription of ISGs.^19^ Subsequently, IL‐29 presented the ability of antiviral protection, anti‐proliferative response, antitumour activities and immune regulation.^15^ All these revealed that IL‐29 may involve in cytokine secretion and regulation of cellular function through modulating the activation and signalling transduction of signalling pathways (Figure 1).

## 3 BIOLOGIC FUNCTIONS OF IL‐29

### 3.1 Effect of IL‐29 on non‐immune cells

Human keratinocytes (KCs) were reported to induce expression of antiviral proteins and TLR3.^18^ Treatment of KCs with IL‐29 up‐regulated 2', 5'‐oligoadenylate synthetase (2', 5'‐OAS) and myxovirus resistance A (MxA) expression. Administration of IL‐29 in KCs also promoted TLR3 expression.^18^ IL‐29 treatment significantly enhanced the expression of IFN‐β induced by herpes simplex virus type 1 (HSV‐1) and protected KCs from HSV‐1 attack.^18^ Therefore, IL‐29 could regulate TLR3 and IFN‐β expression in KCs and then play a part in antiviral activity.

### 3.2 Significant role of IL‐29 in innate immunity

Monocytes respond well to IL‐29 and produce several cytokines. Human monocytes stimulated with IL‐29 significantly up‐regulated levels of IL‐6, IL‐8 and IL‐10. IL‐19 is a member of IL‐10 family cytokine. IL‐29 treatment on monocytes had an ability to enhance levels of IL‐19.^20^ Monocytes morphology changed quickly and became motile with IL‐29 treatment. These data suggested the potential role of IL‐29 in function of monocytes.^20^ IL‐29–stimulated monocyte‐derived macrophage in combination with LPS or TLR7/8 agonist resiquimod (R848) treatment enhanced tumour necrosis factor (TNF) production.^21^ Similarly, IL‐29 could enhance IL‐12p40 expression in human monocyte‐derived macrophage. IL‐29 treatment also up‐regulated TLR8 expression.^21^ IL‐29 stimulation on human monocyte‐derived macrophages led to increased IL‐10–induced pSTAT3 and IL‐10R1 expression, indicating an ability of IL‐29 to augment IL‐10 signalling events in macrophages.^22^ IL‐29 improved cell surface expression of interferon gamma receptor 1 (IFNGR1) on monocyte‐derived macrophage.^17^ In addition, macrophage reacted to IL‐29 promoted the generation of inflammatory cytokines IL‐6, IL‐8 and IL‐10.^20^ Natural killer (NK) cell does not express IFN‐λR1 chain. It is impossible for IL‐29 to affect the cell directly. However, IL‐29 showed an indirect role in cytokine‐dependent manner through the interaction between macrophage and NK cell, where IL‐29 combined with IFN‐γ indirectly influenced NK cell through macrophage‐mediated IL‐12 production, and subsequently elicited IFN‐γ production.^23^ Therefore, IL‐29 indirectly affected NK cells, mediated through the stimulation of macrophages, suggesting that IL‐29 modulates the function of macrophages.

Interleukin‐29–treated plasmacytoid dendritic cells (pDCs) showed reduced expression of IFN‐γ, IL‐13 and IL‐10, and negatively regulated the maturation and activation of pDCs.^24^, ^25^ Stimulation of pDCs with IL‐29 could up‐regulate the expression of CD80 and inducible costimulatory molecule‐L (ICOS‐L), C‐C chemokine receptor type 7 (CCR7) and L‐selectin (CD62L).^25^ IL‐29 in combination with IFN‐α significantly enhanced the expression of the costimulatory molecules CD80, CD83 and ICOS‐L. pDCs are the main secretor of IFN‐α.^26^ IL‐29 combined with cytosine‐phosphate‐guanosine oligodeoxynucleotides (CpG‐ODN) or GPG2216 stimulation on pDCs promoted INF‐α secretion.^26^, ^27^ These data reflected that IL‐29 is able to regulate costimulatory molecules expression and may play a role in activation and immunostimulatory potential of pDCs.

Neutrophils and neutrophil‐released meshwork structures are called extraneutrophil traps (NETs), which are the main mediators and emerging therapeutic targets of thrombosis. Treatment of IL‐29 inhibited NET formation in neutrophils. Amount of cytoplasmic tissue factors in neutrophil was also hindered after IL‐29 stimulation.^28^ In addition, IL‐29 suppressed the neutrophil migratory capacity in prothrombotic and proNETotic functions of neutrophils, indicating the function of IL‐29 in neutrophils, for instance, inhibiting thrombo‐inflammation.

Mast cells expressing IL‐29 are located in some human tissues such as colon, tonsil and lung. Mast cells release IL‐29 under proteolytic allergen stimulation. After stimulating mast cells with IL‐29, levels of IL‐4 and IL‐13 were enhanced.^16^ In addition, exposure of mast cells to IL‐29 up‐regulated the secretion of IL‐6. However, the effect of IL‐29 on inflammatory cytokine generation can be inhibited following adding IL‐29 antibody. Action of tryptic proteases in mast cells is influenced by IL‐29, where IL‐29 down‐regulated expression of protease‐activated receptor (PAR) 1, and up‐regulated expression of PAR2, PAR3 and PAR4.^29^ Injection of IL‐29 in mouse peritoneum induced mast cell accumulation, whereas the accumulation of IL‐29–induced mast cells was abolished after adding Scianna‐2 (SC2) antibody.^16^ These findings suggested that IL‐29 had an ability to regulate cytokine release and induce mast cell infiltration.

### 3.3 IL‐29 plays important roles in adaptive immunity

Human CD27^−^ naive and CD27^+^ B cells stimulated by IL‐29 alone or in combination with the TLR7/8 ligand R848 significantly up‐regulated the marker CD69, indicating that both naive and memory B cell populations were responsive to IL‐29 stimulation.^6^ CD19^+^ B cells stimulated with IL‐29 could significantly up‐regulate IFN‐stimulated genes myxovirus resistance‐1 (Mx1) and OAS1 expression. IL‐29 stimulation on CD19^+^ B cells also up‐regulated TLR7 expression.^6^ Intriguingly, the combination of IL‐29 with R848 enhanced TLR7/8‐mediated IgG and IgM production, and the production of R848‐mediated IL‐6 on treated CD19^+^ B cells, implying the role of IL‐29 to regulate TLR7/8‐triggered cytokine secretion.^6^ Addition of IL‐29 enhanced R848‐induced proliferation of CD19^+^ B cell.^6^ Collectively, these findings suggested IL‐29 to regulate the function of B cells, especially binding to TLR7/8 ligation.

**Figure 2:**
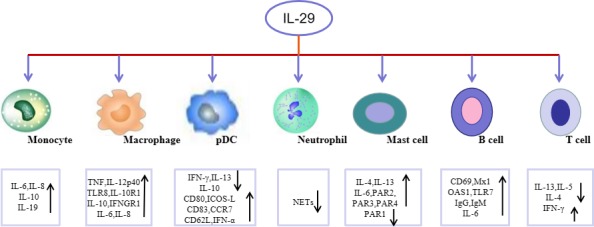
Functional role of IL‐29 in immune cells. IL‐29 regulates the generation of inflammatory cytokines, and chemokines in different immune cells. TNF, tumour necrosis factor; TLR8, toll‐like receptor 8; IFNGR1, interferon gamma receptor 1; IL‐10R1, interleukin‐10 receptor 1; IFN, interferon; ICOS, inducible T cell costimulator; CCR7, C‐C chemokine receptor type 7; CD62L, L‐selectin; NETs, extraneutrophil traps; PAR, protease‐activated receptor; Mx1, myxovirus resistance‐1; OAS1, oligoadenylate synthetase 1; TLR7, toll‐like receptor 7

Interleukin‐29 directly inhibited Th2 polarization by regulating Th2 restrictive transcription factor GATA3.^24^ IL‐4, IL‐5 and IL‐13 are three Th2 cytokines.^30^ IL‐29 treatment on CD4^+^ T cells inhibited the production of IL‐13 and enhanced IFN‐γ (representative of Th1 responses). IL‐4 strongly increased IL‐13 production in naive CD4^+^ T cells, showing that IL‐29 is possible to antagonize the activity of IL‐4 in Th2 response.^24^ IL‐29 receptor IL‐28Rα was expressed on naive and memory CD4^+^ T cells. Naive or memory CD4^+^ T cells under the Th2‐polarizing condition in the presence of IL‐29 significantly reduced the secretion of a Th2 cytokine IL‐5.^24^ Furthermore, stimulation of CD3^+^ CD4^+^ T cells with IL‐29 down‐regulated the levels of IL‐13 and the amount of IL‐13+ CD3^+^ CD4^+^ T cells.^30^ IL‐29 treatment on CD3^+^ CD4^+^ T cells also noted reduction in IL‐4 and IL‐5 production.^30^ Collectively, IL‐29 may affect release of inflammatory cytokines in T cells and regulation of T cell differentiation (Figure 2).

## 4 IL‐29 AND INFLAMMATORY AUTOIMMUNE DISEASES

### 4.1 IL‐29 expression in inflammatory autoimmune disorders

Rheumatoid arthritis is a type of chronic systemic autoimmune disease. IL‐29 was expressed in CD68^+^ macrophage and FGF‐2+ fibroblast in the lining layers of RA synovium.^31^ Expression of IL‐29 was significantly higher in serum, peripheral blood mononuclear cells (PBMCs) and synovial tissue in RA patients compared with that in healthy controls.^11^, ^32^ Levels of IL‐29 in synovial fluid (SF) were higher in RA patients compared with that in OA patients.^31^ Serum levels of IL‐29 positively correlated with rheumatoid factor (RF), anti‐cyclic citrullinated peptide (anti‐CCP) antibody and disease activity score of 28. Interestingly, anti–CCP‐positive RA patients had higher serum IL‐29 levels than that in healthy controls and anti–CCP‐negative RA patients.^32^ In RA synovial fluid, increased level of granzyme M (GrM) could induce the release of IL‐29.^33^ In SLE patients, IL‐29 expression was increased in SLE patients as compared to healthy controls. SLE patients with active disease showed elevated expression of IL‐29 than the less active group, suggesting that IL‐29 may imply disease activity.^34^ SLE patients sometimes showed clinical manifestations, such as alopecia, mucosal ulcer, malar rash, chest affection and thrombocytopenia.^35^ SLE patients with renal disease and arthritis in contrast to patients without the manifestations showed increased serum IL‐29 levels.^34^ Serum levels of IL‐29 correlated with SLE disease activity index, anti‐dsDNA antibody and C‐reactive protein.^34^ In OA patients, IL‐29 expression was strongly higher in OA PBMCs than healthy controls. Serum levels of IL‐29 and IL‐29 expression in synovium were elevated in OA patients when compared to healthy controls.^15^ Therefore, expression of IL‐29 was dysregulated in RA, SLE and OA patients, and correlated with disease activity.

Levels of IL‐29 were strongly increased in serum of SS patients with intermediate minor salivary gland tissue (MSG) infiltrates when compared to the controls.^13^ Psoriasis and atopic dermatitis (AD) are two common chronic inflammatory skin diseases. IL‐29 can be derived from Th17 cells, and IL‐29 expression was enhanced in lesion skin of psoriasis patients.^36^ Levels of IL‐29 in psoriatic lesions positively correlated with antiviral protein expression.^12^ Similarly, serum levels of IL‐29 were elevated in psoriasis patients as compared to healthy controls.^37^ IL‐29 levels were significantly higher in the skin of AD patients when compared to healthy controls.^38^ Hashimoto's thyroiditis (HT) is a typical autoimmune thyroid disease. Serum levels of IL‐29 were higher in HT patients as compared to healthy controls.^39^ Furthermore, systemic sclerosis (SSc) patients showed increased IL‐29 serum levels as compared to that in healthy controls.^40^ Childhood uveitis is a sight‐threatening inflammatory eye disease. Most patients with uveitis are associated with juvenile idiopathic arthritis (JIA). IL‐29 levels were down‐regulated in ocular fluid from JIA‐associated uveitis patients. IL‐29 intraocular levels were decreased in JIA‐associated uveitis patients when compared to those with idiopathic uveitis.^41^ Taken together, IL‐29 levels were abnormal in some other inflammatory autoimmune diseases including SS, psoriasis, AD, HT, SSc and uveitis (Table 1).

**Table 1:** Abnormal expression of IL‐29 in inflammatory autoimmune diseases

Disorder name	Expression of IL‐29	References
Rheumatoid arthritis	Serum, synovial tissue^a^, PBMCs^b^	11↑^31^↑^32^↑^33^↑
Systemic lupus erythematosus	Serum	34↑
Osteoarthritis	Serum, synovium^c^, PBMCs^d^	15↑
Sjögren's syndrome	Serum	13↑
Psoriasis	Serum, lesional skin^a^	36↑^37^↑
Atopic dermatitis	Skin	38↑
Hashimoto's thyroiditis	Serum	39↑
Systemic sclerosis	Serum	40↑
Uveitis	Ocular fluid	41↓

a Increased levels of IL‐29 were detected in serum and lesion skin of psoriasis patients.

^a,b^ Rheumatoid arthritis patients showed elevated expression of IL‐29 in serum, synovial tissue and peripheral blood mononuclear cells (PBMCs).

^c,d^ The levels of IL‐29 were up‐regulated in serum, synovium and PBMCs of osteoarthritis patients.

### 4.2 IL‐29 regulates the production of autoimmune‐related components

TLR4 plays an important role in synovial inflammation and contributes to the pathogenesis of RA.^42^ Expression of TLR4 in RAW264.7 cells was increased when exposed to IL‐29.^11^ IL‐6, IL‐8 and MMP‐3 protein levels were significantly enhanced in RA fibroblast cell line MH7A by IL‐29 stimulation, whereas IL‐29 exposure down‐regulated IL‐10 expression. Administration of IL‐29 further promoted the TLR4‐mediated IL‐6 and IL‐8 expression in RA arthritis synovial fibroblasts.^11^ Similarly, IL‐29 up‐regulated TLR2, TLR3 and TLR4 expression in RA FLS.^43^, ^44^ Chronic inflammation is an important factor of ongoing cartilage damage and joint degeneration in OA pathogenesis. IL‐29 was reported to induce the up‐regulation of MMP‐1/TIMP‐1, MMP‐2/TIMP‐1, MMP‐3/TIMP‐1 and MMP‐13/TIMP‐1 ratio in OA FLS. Levels of IL‐1β, IL‐6, IL‐8 and MMP‐3 in OA FLS were up‐regulated by IL‐29 incubation. On the contrary, the effect of IL‐29 on thee cytokines was disrupted by usage of IL‐29 blocking antibody.^15^ IL‐29 treatment on psoriatic mouse skin showed increased expression of C‐X‐C motif chemokine 10 (CXCL10) and CXCL11, provoked T cell infiltration and skin swelling.^36^ Biopsies obtained from the lesion of psoriasis patients under the condition of anti–IL‐29 antibody treatment down‐regulated the expression of antiviral proteins.^12^ Overall, above findings provided clues that IL‐29 may involve in the pathogenesis of inflammatory autoimmune diseases by regulating expression of the inflammatory components.

### 4.3 Therapeutic potential of IL‐29

Previous investigations have confirmed that IL‐29 plays a vital role in several therapeutic fields, including hepatitis C and B, and melanoma, due to its ability of antiviral and antitumour.^3^, ^27^ In recent years, attention has been paid to the role of this cytokine in immune regulation. IL‐29 is abnormally expressed in some inflammatory autoimmune diseases. Moreover, numerous researches have shown that IL‐29–mediated inflammation plays an important role in the pathogenesis of these diseases. Given the biological role of IL‐29 in many immune and non‐immune cells, including KCs, monocytes, pDC, B cells, mast cells and its regulatory ability in many autoimmune components, this cytokine may be considered as a therapeutic target for the complex inflammatory diseases such as OA and RA. However, more information about the application of IL‐29 in the treatment of inflammatory autoimmune disorders needs to be further clarified.

## 5 REGULATION OF IL‐29 EXPRESSION

Above findings showed that IL‐29 regulates the production of inflammatory cytokines and chemokines, and therefore may play a potential role in inflammatory autoimmune disease. In turn, regulation of IL‐29 expression may reverse the abnormal expression and function of IL‐29, giving the possibility to inhibit the generation of inflammatory components and suppress the development of the disorders. In normal conditions, pDCs treated with HSV or imiquimod produced high levels of IL‐29.^25^ IL‐29 is known to induce IL‐12p40 production in macrophages. However, the effect can be inhibited in the presence of IFN‐α.^21^ Exposure of P815 cells to SC2 antibody hindered the capacity of IL‐29 to induce IL‐4, IL‐13 and IL‐6 release.^29^ After adding rPer, a common allergen, the levels of IL‐29 in HMC‐I cell were increased. Interestingly, the effect of rPer on IL‐29 expression in HMC‐1 cell was suppressed by adding PA1 blocking antibody. Poly(I:C) induced significant up‐regulation of IL‐29 expression.^16^ Studies have shown that IL‐29 makes a significant effect on the Th2 response via antagonizing the effect of CD4^+^ T cells and Th2‐related cytokine production. Nevertheless, production of IFN‐λ in turn was controlled by Th2 cytokines.^24^ IL‐29 was able to regulate IL‐13 release, but was abolished following adding proteinase‐K in naive CD3^+^ T cells.^45^ When stimulated PBMCs from volunteers with IL‐4, transcription and secretion of IL‐29 were increased.^46^ IL‐29 is recognized to regulate IL‐19 expression. However, the capacity of IL‐29 to induce IL‐19 was hindered by IL‐10 in PBMCs.^20^ When stimulated human fibroblasts with purified granzymes, expression of IL‐29 was increased.^33^ These data indicated that IL‐29 can be regulated by different stimulation in normal conditions, such as IFN‐α and Th2‐related cytokines. In autoimmunity, it was accepted that IL‐29 secretion was inhibited by IL‐4 stimulation on Th17 cells from psoriasis patients.^12^ In arthritis, the role of IL‐29 to enhance the expression of TLR4 in macrophage cell line RAW264.7 cells could be strengthened after adding LPS stimulation. Activation of NF‐κB signalling became more significant when RAW264.7 cells stimulated with both IL‐29 and LPS than IL‐29 stimulation alone, suggesting that LPS may regulate the effect of IL‐29 and the role of IL‐29.^11^ In RA patients, serum IL‐29 levels were reduced after the treatment with DMARDs.^32^ Collectively, these data suggested that IL‐29 is also controlled in autoimmunity.

## 6 CONCLUSION

Interleukin‐29 is a newly discovered cytokine and belongs to the IL‐10 family. This cytokine has become a research hotspot recently. Compared to IL‐28A and IL‐28B, IL‐29 is more structurally unique. Furthermore, IL‐29 is considered to be an effective IFN molecule in human and seems to be the most abundant IFN molecule in serum.^47^ Findings from animal models and human have both proved the important role of IL‐29 in inflammation.^31^, ^32^, ^34^, ^35^, ^43^, ^44^ For example, TLR7/8‐mediated B cell proliferation and IgG production are enhanced via IL‐29 stimulation. IL‐29 is able to inhibit T cell differentiation. Moreover, IL‐29 can enhance the IL‐10 signalling events in macrophage and plays a vital role in modulating the function of monocyte‐derived macrophage. IL‐29 signals via binding to receptors, including IL‐28Rα and IL‐10R2. Inflammatory autoimmune diseases are a group of diseases, such as SLE, RA and OA. These kinds of diseases are characterized by damage and dysfunction of specific or multiple organs and tissues.^48^, ^49^ The present study reviewed much findings that discussed the role of IL‐29 in inflammatory autoimmune disorders, suggesting the therapeutic potential of IL‐29 in inflammatory diseases. However, the pathogenesis of inflammatory autoimmune diseases is complex. Therefore, further studies regarding the clear role and therapeutic effects of IL‐29 are required.

## CONFLICT OF INTEREST

All the authors who have taken part in this study declared that they have no conflicts of interest to this manuscript.

## AUTHOR CONTRIBUTIONS

J. M. W contributed to the literature search and manuscript editing. A. F. H drew pictures and edited tables. W. D. X and L. C. S contributed to the reviewing of the manuscript.

## Data Availability

Data are available.

## References

[1] Hosokawa Y , Hosokawa I , Shindo S , et al. IL‐29 enhances CXCL10 production in TNF‐α‐stimulated human oral epithelial cells. Immunol Invest. 2017;46(6):615‐624. 28753407 10.1080/08820139.2017.1336176

[2] Meager A , Heath A , Dilger P , et al. Standardization of human IL‐29 (IFN‐λ1): establishment of a World Health Organization international reference reagent for IL‐29 (IFN‐λ1). J Interferon Cytokine Res. 2014;34(11):876‐884. 24955567 10.1089/jir.2014.0015 PMC4216994

[3] Witte K , Witte E , Sabat R , et al. IL‐28A, IL‐28B, and IL‐29: promising cytokines with type I interferon‐like properties. Cytokine Growth Factor Rev. 2010;21(4):237‐251. 20655797 10.1016/j.cytogfr.2010.04.002

[4] Ouyang W , Rutz S , Crellin NK , et al. Regulation and functions of the IL‐10 family of cytokines in inflammation and disease. Annu Rev Immunol. 2011;29:71‐109. 21166540 10.1146/annurev-immunol-031210-101312

[5] Yu D , Zhao M , Dong L , et al. Design and evaluation of novel interferon lambda analogs with enhanced antiviral activity and improved drug attributes. Drug Des Devel Ther. 2016;10:163‐182. 10.2147/DDDT.S91455 PMC4708225 26792983

[6] de Groen RA , Groothuismink ZM , Liu BS , et al. IFN‐λ is able to augment TLR‐mediated activation and subsequent function of primary human B cells. J Leukoc Biol. 2015;98(4):623‐630. 26130701 10.1189/jlb.3A0215-041RR

[7] Sommereyns C , Paul S , Staeheli P , et al. IFN‐lambda (IFN‐lambda) is expressed in a tissue‐dependent fashion and primarily acts on epithelial cells in vivo. PLoS Pathog. 2008;4(3):e1000017. 18369468 10.1371/journal.ppat.1000017 PMC2265414

[8] Guenterberg KD , Grignol VP , Raig ET , et al. Interleukin‐29 binds to melanoma cells inducing Jak‐STAT signal transduction and apoptosis. Mol Cancer Ther. 2010;9(2):510‐520. 20103601 10.1158/1535-7163.MCT-09-0461 PMC2820597

[9] Peng Q , Luo A , Zhou Z , et al. Interleukin 29 inhibits RANKL‐induced osteoclastogenesis via activation of JNK and STAT, and inhibition of NF‐κB and NFATc1. Cytokine. 2019;113:144‐154. 30001863 10.1016/j.cyto.2018.06.032

[10] Lin SC , Kuo CC , Tsao JT , et al. Profiling the expression of interleukin (IL)‐28 and IL‐28 receptor α in systemic lupuserythematosus patients. Eur J Clin Invest. 2012;42(1):61‐69. 21707611 10.1111/j.1365-2362.2011.02557.x

[11] Xu D , Yan S , Wang H , et al. IL‐29 enhances LPS/TLR4‐mediated inflammation in rheumatoid arthritis. Cell Physiol Biochem. 2015;37(1):27‐34. 26278073 10.1159/000430330

[12] Wolk K , Witte K , Witte E , et al. IL‐29 is produced by T(H)17 cells and mediates the cutaneous antiviral competence in psoriasis. Sci Transl Med. 2013;5(204):204ra129. 10.1126/scitranslmed.3006245 24068736

[13] Apostolou E , Kapsogeorgou EK , Konsta OD , et al. Expression of type III interferons (IFNλs) and their receptor in Sjögren's syndrome. Clin Exp Immunol. 2016;186(3):304‐312. 27613139 10.1111/cei.12865 PMC5108072

[14] Kelm NE , Zhu Z , Ding VA , et al. The role of IL‐29 in immunity and cancer. Crit Rev Oncol Hematol. 2016;106:91‐98. 27637354 10.1016/j.critrevonc.2016.08.002 PMC7129698

[15] Xu L , Peng Q , Xuan W , et al. Interleukin‐29 enhances synovial inflammation and cartilage degradation in osteoarthritis. Mediators Inflamm. 2016;2016:9631510. 27433031 10.1155/2016/9631510 PMC4940582

[16] He S , Zhang H , Chen H , et al. Expression and release of IL‐29 by mast cells and modulation of mast cell behavior by IL‐29. Allergy. 2010;65(10):1234‐1241. 20337614 10.1111/j.1398-9995.2010.02349.x

[17] Boisvert M , Shoukry NH . Type III interferons in hepatitis C virus infection. Front Immunol. 2016;7:628. 28066437 10.3389/fimmu.2016.00628 PMC5179541

[18] Zhang SQ , Zhang Z , Luo X , et al. Interleukin 29 enhances expression of Toll receptor 3 and mediates antiviral signals in humankeratinocytes. Inflamm Res. 2011;60(11):1031‐1037. 21847628 10.1007/s00011-011-0364-z

[19] Li Q , Kawamura K , Tada Y , et al. Novel type III interferons produce anti‐tumor effects through multiple functions. Front Biosci (Landmark Ed). 2013;18:909‐918. 23747856 10.2741/4152

[20] Jordan WJ , Eskdale J , Boniotto M , et al. Modulation of the human cytokine response by interferon lambda‐1 (IFN‐lambda1/IL‐29). Genes Immun. 2007;8(1):13‐20. 17082759 10.1038/sj.gene.6364348

[21] Liu BS , Janssen HL , Boonstra A . IL‐29 and IFNα differ in their ability to modulate IL‐12 production by TLR‐activated humanmacrophages and exhibit differential regulation of the IFNγ receptor expression. Blood. 2011;117(8):2385‐2395. 21190998 10.1182/blood-2010-07-298976

[22] Liu BS , Janssen HL , Boonstra A . Type I and III interferons enhance IL‐10R expression on human monocytes and macrophages, resulting in IL‐10‐mediated suppression of TLR‐induced IL‐12. Eur J Immunol. 2012;42(9):2431‐2440. 22685028 10.1002/eji.201142360

[23] de Groen RA , Boltjes A , Hou J , et al. IFN‐λ‐mediated IL‐12 production in macrophages induces IFN‐γ production in human NK cells. Eur J Immunol. 2015;45(1):250‐259. 25316442 10.1002/eji.201444903

[24] Dai J , Megjugorac NJ , Gallagher GE , et al. IFN‐lambda1 (IL‐29) inhibits GATA3 expression and suppresses Th2 responses in human naive and memory T cells. Blood. 2009;113(23):5829‐5838. 19346497 10.1182/blood-2008-09-179507

[25] Megjugorac NJ , Gallagher GE , Gallagher G . Modulation of human plasmacytoid DC function by IFN‐lambda1 (IL‐29). J Leukoc Biol. 2009;86(6):1359‐1363. 19759281 10.1189/jlb.0509347

[26] Cho CH , Yoon SY , Lee CK , et al. Effect of interleukin‐29 on interferon‐α secretion by peripheral blood mononuclear cells. Cell J. 2015;16(4):528‐537. 25685743 10.22074/cellj.2015.497 PMC4297491

[27] Wisgrill L , Wessely I , Netzl A , et al. Diminished secretion and function of IL‐29 is associated with impaired IFN‐α response of neonatal plasmacytoid dendritic cells. J Leukoc Biol. 2019. 10.1002/JLB.4A0518-189R PMC6852569 31211458

[28] Chrysanthopoulou A , Kambas K , Stakos D , et al. Interferon lambda1/IL‐29 and inorganic polyphosphate are novel regulators of neutrophil‐driven thromboinflammation. J Pathol. 2017;243(1):111‐122. 28678391 10.1002/path.4935

[29] Zhang H , Yang H , Ma W , et al. Modulation of PAR expression and tryptic enzyme induced IL‐4 production in mast cells by IL‐29. Cytokine. 2013;61(2):469‐477. 23218741 10.1016/j.cyto.2012.10.032

[30] Srinivas S , Dai J , Eskdale J , et al. Interferon‐lambda1 (interleukin‐29) preferentially down‐regulates interleukin‐13 over other T helper type 2 cytokine responses in vitro. Immunology. 2008;125(4):492‐502. 18547367 10.1111/j.1365-2567.2008.02862.x PMC2612545

[31] Wang F , Xu L , Feng X , et al. Interleukin‐29 modulates proinflammatory cytokine production in synovialinflammation of rheumatoid arthritis. Arthritis Res Ther. 2012;14(5):R228. 23078630 10.1186/ar4067 PMC3580539

[32] Chang QJ , Lv C , Zhao F , et al. Elevated serum levels of interleukin‐29 are associated with disease activity in rheumatoid arthritis patients with anti‐cyclic citrullinated peptide antibodies. Tohoku J Exp Med. 2017;241(2):89‐95. 28154345 10.1620/tjem.241.89

[33] Shan L , van den Hoogen LL , Meeldijk J , et al. Increased intra‐articular granzyme M may trigger local IFN‐λ1/IL‐29 response in rheumatoid arthritis. Clin Exp Rheumatol. 2019. 31172927

[34] Wu Q , Yang Q , Lourenco E , et al. Interferon‐lambda1 induces peripheral blood mononuclear cell‐derived chemokines secretion in patients with systemic lupus erythematosus: its correlation with disease activity. Arthritis Res Ther. 2011;13(3):R88. 21679442 10.1186/ar3363 PMC3218903

[35] Xu WD , Su LC , Xie QB , et al. Interleukin‐2‐inducible T‐cell kinase expression and relation to disease severity in systemic lupus erythematosus. Clin Chim Acta. 2016;463:11‐17. 27729219 10.1016/j.cca.2016.10.010

[36] Witte E , Kokolakis G , Witte K , et al. Interleukin‐29 induces epithelial production of CXCR3A ligands and T‐cell infiltration. J Mol Med (Berl). 2016;94(4):391‐400. 26612594 10.1007/s00109-015-1367-y

[37] Cardoso PR , Lima EV , Lima MM , et al. Clinical and cytokine profile evaluation in Northeast Brazilian psoriasisplaque‐type patients. Eur Cytokine Netw. 2016;27(1):1‐5. 27094154 10.1684/ecn.2016.0371

[38] Fedenko ES , Elisyutina OG , Filimonova TM , et al. Cytokine gene expression in the skin and peripheral blood of atopic dermatitis patients and healthy individuals. Self Nonself. 2011;2(2):120‐124. 22299064 10.4161/self.2.2.16939 PMC3268998

[39] Arpaci D , Karakas Celik S , Can M , et al. Increased serum levels of IL‐28 and IL‐29 and the protective effect of IL28B rs8099917 polymorphism in patients with hashimoto's thyroiditis. Immunol Invest. 2016;45(7):668‐678. 27617784 10.1080/08820139.2016.1208215

[40] Dantas AT , Gonçalves SM , Pereira MC , et al. Interferons and systemic sclerosis: correlation between interferon gamma and interferon‐lambda 1 (IL‐29). Autoimmunity. 2015;48(7):429‐433. 26057401 10.3109/08916934.2015.1054028

[41] Haasnoot AM , Kuiper JJ , Hiddingh S , et al. Ocular fluid analysis in children reveals interleukin‐29/Interferon‐λ1 as a biomarker for juvenile idiopathic arthritis‐associated uveitis. Arthritis Rheumatol. 2016;68(7):1769‐1779. 26866822 10.1002/art.39621

[42] Liu Y , Yin H , Zhao M , et al. TLR2 and TLR4 in autoimmune diseases: a comprehensive review. Clin Rev Allergy Immunol. 2014;47(2):136‐147. 24352680 10.1007/s12016-013-8402-y

[43] Wang F , Xu L , Feng X , et al. Interleukin‐29 modulates proinflammatory cytokine production in synovial inflammation of rheumatoid arthritis. Arthritis Res Ther. 2012;14(5):R228. 23078630 10.1186/ar4067 PMC3580539

[44] Xu L , Feng X , Tan W , et al. IL‐29 enhances Toll‐like receptor‐mediated IL‐6 and IL‐8 production by the synovial fibroblasts from rheumatoid arthritis patients. Arthritis Res Ther. 2013;15(5):R170. 24286242 10.1186/ar4357 PMC3978693

[45] Jordan WJ , Eskdale J , Srinivas S , et al. Human interferon lambda‐1 (IFN‐lambda1/IL‐29) modulates the Th1/Th2 response. Genes Immun. 2007;8(3):254‐261. 17361203 10.1038/sj.gene.6364382

[46] Megjugorac NJ , Gallagher GE , Gallagher G . IL‐4 enhances IFN‐lambda1 (IL‐29) production by plasmacytoid DCs via monocyte secretion of IL‐1Ra. Blood. 2010;115(21):4185‐4190. 20233967 10.1182/blood-2009-09-246157

[47] Alborzi AM , Bamdad T , Davoodian P , et al. Insights into the role of HCV Plus‐/Minus strand RNA, IFN‐γ and IL‐29 in relapse outcome in patients infected with HCV. Asian Pac J Allergy Immunol. 2015;33(3):173‐181. 26342113 10.12932/AP0570.33.3.2015

[48] Gravina G , Wasén C , Garcia‐Bonete MJ , et al. Survivin in autoimmune diseases. Autoimmun Rev. 2017;16(8):845‐855. 28564620 10.1016/j.autrev.2017.05.016

[49] Toubi E , Vadasz Z . Innate immune‐responses and their role in driving autoimmunity. Autoimmun Rev. 2019;18(3):306‐311. 30639645 10.1016/j.autrev.2018.10.005

